# Can the First Fasting Plasma Glucose Test in Pregnancy Predict Subsequent Gestational Complications?

**DOI:** 10.1155/2022/9633664

**Published:** 2022-04-11

**Authors:** Silvia Burlina, Maria Grazia Dalfrà, Pietro Belloni, Serena Ottanelli, Federico Mecacci, Giorgio Mello, Annunziata Lapolla

**Affiliations:** ^1^Department of Medicine—DIMED, University of Padova, Padua, Italy; ^2^Department of Statistical Sciences, University of Padua, Padua, Italy; ^3^Department of Experimental, Clinical and Biochemical Science, University of Florence, Florence, Italy

## Abstract

**Objective:**

To determine the best cut-off level of pregnant women's first fasting plasma glucose (FFPG) test results for the prediction of subsequent onset of gestational diabetes mellitus (GDM) and to examine the association between FFPG and maternal and neonatal outcomes in a large Caucasian population.

**Methods:**

1437 medical records of women with singleton pregnancies followed up between 2015 and 2018 were retrospectively analyzed. Data on FFPG tested in the first trimester and 75 g oral glucose tolerance test (OGTT) findings performed according to IADPSG criteria and Italian guidelines were collected and evaluated. The women's clinical and metabolic characteristics (age, prepregnancy body mass index (BMI), previous pregnancies complicated by GDM, timing of delivery, and gestational hypertension) were also recorded. The fetal variables considered were being large for gestational age (LGA) or small for gestational age (SGA), macrosomia, and hypoglycemia.

**Results:**

Among the 1437 pregnant women studied, 684 had a normal glucose tolerance (NGT) and 753 developed GDM. In a univariate analysis FFPG ≥92 mg/dl predicts the risk of GDM with an OR = 2.36 (95% CI 1.930–3.186; *p* < 0.001). In multivariate analysis, after adjusting for principal risk factors of GDM (BMI, previous GDM, age >35 years, family history of diabetes) FFPG ≥92 mg/dl was associated with the risk of GDM (OR = 1.92; 95% CI 1.488–2.492; *p* < 0.001). In univariate analysis, FFPG ≥92 mg/dl predict the risk of insulin therapy in GDM women with a OR = 1.88 (95% CI 1.230–2.066; *p* < 0.001). As regards LGA, in a multivariate analysis, after adjusting for BMI, FFPG ≥92 mg/dl was associated with the risk of LGA only in NGT women (OR = 2.34; 95% CI 1.173–4.574; *p*=0.014), but not in GDM women. FFPG was not associated with other maternal or neonatal outcomes.

**Conclusions:**

FFPG ≥92 mg/dl is related to GDM diagnosis and to the need of insulin therapy if GDM is diagnosed. An early diagnosis and a prompt start of insulin therapy are essential to prevent maternal and fetal complications.

## 1. Introduction

Gestational diabetes mellitus (GDM) is now defined as a “hyperglycemia diagnosed in the second or third trimester of pregnancy that is not clearly overt diabetes” [1]. The international guidelines have addressed the importance of diagnosing GDM as soon as possible due to the frequent adverse maternal and fetal outcomes related to this condition if it goes untreated [1–3]. Identifying women at higher risk of GDM early in their pregnancy is important because it has been demonstrated that an appropriate diet, exercise and medication positively affect maternal and fetal outcomes [4, 5].

In principle, it is feasible to perform a fasting plasma glucose (FPG) test early in pregnancy as a screening test to identify women at risk of GDM. FPG is easy to measure, inexpensive and reproducible. What FPG level to consider as normal in pregnancy is still debated, however. According to the IADPSG guidelines, FPG levels from 92 to 125 mg/dl at the first prenatal visit are diagnostic of GDM [2]. These values were extrapolated from the cutoff used to diagnose GDM later in a pregnancy, but it is common knowledge that there is a physiological drop in FPG early in a normal pregnancy.

A strong correlation has been demonstrated between hyperglycemia at the beginning of a pregnancy (even with blood sugar levels in a range not diagnostic for diabetes) and an increased risk of adverse outcomes [6]. Bianchi et al. demonstrated that 80% of pregnant women with FPG levels at first trimester from 100 to 125 mg/dl progress to GDM unless preventive action is taken [7]. In addition, Zhu et al. demonstrated that incidence of GDM was 66.2% for women with FPG >110 mg/dl at first prenatal visit [8] and Harrison et al. demonstrated that FPG levels between 88 and 120 mg/dl at first trimester was the strongest predictor of GDM development [9].

Hence our interest in retrospectively determining in a large Caucasian population the best cut-off level of pregnant women's first fasting plasma glucose (FFPG) test results for the onset of GDM later in their gestation as well as in examining the association between FFPG and maternal and neonatal outcomes.

## 2. Materials and Methods

This retrospective study analyzed 1437 medical records of women with singleton pregnancies followed up at the Diabetology Unit of ULSS6 Padova University and the Gynecology and Obstetrics Unit at the Careggi Hospital in Florence, between 2015 and 2018.

This study was conducted in accordance with standards of the 1975 Helsinki Declaration and its later amendments and was approved by the local ethic committee.

All pregnant women were screened and diagnosed according to IADPSG criteria [2] and Italian guidelines [10]. They underwent fasting plasma glucose testing in the first trimester of pregnancy and, if the result was normal (<100 mg/dl), an oral glucose tolerance test (OGTT) was performed at 24–28 weeks of gestation (WG). In women at high risk of GDM (BMI ≥30 kg/m^2^, previous GDM, fasting glucose levels between 100 and 125 mg/dl in the first trimester), the OGTT was performed at 16–18 WG. Women found negative at the OGTT served as controls for the purposes of the present analysis.

Using IADPSG criteria (75 g glucose tolerance test, measuring plasma glucose at 0′, 60′ 120′ after ingesting the glucose), women were classified as having GDM when one or more values exceeded the respective thresholds (<92 mg/dl at the baseline; <180 mg/dl at 60′, <153 mg/dl at 120′) [2].

Pregnant women with type 1 or type 2 diabetes, an impaired glucose tolerance before pregnancy, or a diagnosis of overt diabetes in the first trimester [1] were excluded from the study as well as pregnant women with multiple pregnancies and pregnancies by means of in vitro fertilization or gonadotropin ovulation induction.

Women diagnosed with GDM at their first appointment and at follow-up visits to the Diabetology Unit received multidisciplinary consultant-led care from a diabetologist, a dietician, and a nurse.

They were given an individualized diet, trained to self-monitor their blood glucose (fasting and 1 h after breakfast, lunch, and dinner), and periodically underwent clinical examinations and biochemical tests, as recommended by national guidelines [10]. Insulin treatment was started if they did not meet the treatment goals indicated in the ADA recommendations [11].

The women's clinical and metabolic characteristics were recorded, i.e. age, prepregnancy body mass index (BMI), previous pregnancy complicated by GDM, OGTT results, timing of delivery, maternal morbidities (gestational hypertension). The fetal variables considered were birth weight, macrosomia, and hypoglycemia. Neonates were classified as LGA if their birthweight was above the 90^th^ centile, and small for gestational age if it was below the 10^th^ centile [12]. Macrosomia was defined as a birthweight of 4000 g or more [13]. Neonatal hypoglycemia was defined as glycemia <40 mg/dl in the first 4 hours and/or glycemia <45 in the first 4–24 hours [14].

### 2.1. Statistical Analyses

Data are expressed as means ± SD for continuous variables and percentages for categorical variables. Groups were compared using the *T*-test for unpaired data, and the *χ*^2^ for percentages. A univariable receiver operating characteristic curve (ROC) was computed in order to establish the best cut-off level of the FFPG in predicting development of GDM. A logistic regression was performed to identify parameters capable of predicting GDM. A value <0.05 (two-tailed) was considered significant. The statistical analysis was performed using the statistical software R ver. 3.6.2.

## 3. Results

Among the 1437 pregnant women retrospectively assessed, 684 had a normal glucose tolerance (NGT) and 753 developed GDM.

**Figure 1:**
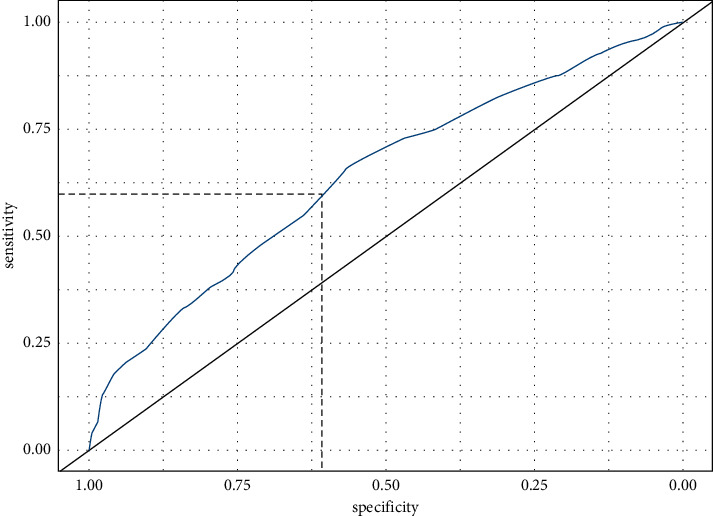
ROC curve for FFPG = 92 mg/dl after logistic regression versus diagnosis of GDM (AUC = 0.64; sensitivity = 59.57% (95% CI: 59.37%—59.75); specificity = 60.71 (95% CI: 60.52%–60.89%)).

We computed a ROC curve to identify the best glucose level for predicting the onset of GDM (Figure 1). The ROC curve indicated that 92 mg/dl was the best cutoff for this purpose (AUC = 0.64), with a sensitivity of 59.57% (95% CI: 59.37%—59.75) and a specificity of 60.71 (95% CI: 60.52%–60.89%). To ascertain the role of FFPG in predicting GDM and pregnancy outcomes, the women with NGT and GDM were both grouped by FFPG: <92 mg/dl or ≥92 mg/dl.

Table 1 shows the clinical and metabolic parameters of the women in our sample, by diagnosis (GDM or NGT) and FFPG findings. Women with a NGT and FFPG ≥92 mg/dl had a significantly higher BMI than women whose FFPG was <92 mg/dl but there were no significant difference in terms of neonatal and maternal outcomes.

Among the women who developed GDM, those with FFPG ≥92 mg/dl were significantly more likely to have a family history of diabetes (*p* < 0.001), a previous GDM diagnosis (*p* < 0.001), were more likely to be more than 35 years old (*p* < 0.001), and to have been obese (*p* < 0.001), or overweight (*p* < 0.001) before becoming pregnant. They also had significantly higher BMI (*p* < 0.001).

GDM women with FFPG ≥92 mg/dl were also diagnosed with GDM significantly earlier in their pregnancy (*p* < 0.001), and required significantly more insulin therapy (*p* < 0.001), by comparison with GDM women whose FFPG was <92 mg/dl.

As concerns neonatal complications, the offspring of GDM women with FFPG ≥92 mg/dl were more likely LGA at limits of significance (*p*=0.0537), while there were no differences in the rates of macrosomia or neonatal hypoglycemia between these two groups. Regarding maternal complications, no significant difference was found between GDM women with FFPG ≥92 mg/dl and those with FFPG <92 mg/dl.

In the comparison between the NGT women with FFPG ≥92 mg/dl and the GDM women with FFPG ≥92 mg/dl, on the other hand, it was among the former that there were more cases of prepregnancy obesity (*p* < 0.001), previous GDM (*p* < 0.001), family history of diabetes (*p* < 0.001) and age >35 years (*p* < 0.05).

In a univariate analysis FFPG ≥92 mg/dl predicts the risk of GDM with an OR = 2.36 (95% CI 1.930–3.186; *p* < 0.001). In multivariate analysis, after adjusting for principle risk factors of GDM (BMI, previous GDM, age >35 years, family history of diabetes) FFPG ≥92 mg/dl was associated with the risk of GDM (OR = 1.92; 95% CI 1.488–2.492; *p* < 0.001), as shown in Table 2.

Then we evaluated FFPG ≥92 mg/dl as a predictor of pregnancy outcomes.

In a univariate analysis, FFPG ≥92 mg/dl predicts the risk of insulin therapy in GDM women with a OR = 1.88 (95% CI 1.230–2.066; *p* < 0.001).

As regards LGA, in a multivariate analysis, after adjusting for BMI, FFPG ≥92 mg/dl was associated with the risk of LGA only in NGT women (OR = 2.34; 95% CI 1.173–4.574; *p*=0.014), but not in GDM women, as shown in Table 3.

## 4. Discussion

The results of our study on a large sample of Caucasian women indicates that FFPG measurements in pregnancy exceeding 92 mg/dl predicts the subsequent onset of GDM.

Several other publications in the literature show that the efficacy of FFPG as a screening test for GDM is debatable. In a high-risk population, Agarwal found FPG unable to diagnose GDM at the first prenatal visit due to a limited specificity [15]. Corrado et al. [16] found that an FPG ≥92 mg/dl could predict GDM later in pregnancy, and Riskin-Mashiah et al. [17] found that the optimal threshold value of FFPG for the prediction of GDM is 83 mg/dl. When Zhu et al. [8] examined 17, 186 medical records of pregnant women attending prenatal clinics in China, they judged that a FPG of 110–124 mg/dl could be used to diagnose GDM at the first prenatal visit. Popova et al. [18] analyzed the medical records of 1584 pregnant women, and found that only the highest glucose levels (>126 mg/dl) at the first prenatal visit were strongly associated with some adverse pregnancy outcomes. It should be noted that different studies used different tests to diagnose GDM (100 g OGTT or 75 g OGTT), and this can partly explain the different conclusions they reached.

In our study, among the women who developed GDM, those with FFPG ≥92 mg/dl were diagnosed significantly earlier than those with FFPG <92 mg/dl. This result underlines the importance of FFPG for the early diagnosis of GDM and supports the Italian Guidelines for the diagnosis of GDM that take into consideration FFPG as risk factor [10]. An early diagnosis and treatment of GDM are essential to preventing maternal and fetal complications [4, 5]. With this in mind, our results showed that a FFPG ≥92 mg/dl could have a value for the purpose of predicting GDM, but it could also be an important factor for clinicians to consider when screening pregnant women for an early identification and treatment of GDM. It is to underline that this is a lower threshold than Italian and international guidelines to define women at high risk of developing GDM. In particular, for Italian guidelines the threshold is a FFPG of 100 mg/dl in the first trimester and for ADA guidelines it is 110 mg/dl prior to 18 weeks of gestation. It will be necessary to conduct prospective studies to better define the threshold of FFPG to determine women at high risk of developing GDM and so at high risk of maternal and fetal complications [1, 10].

Mills et al. demonstrated that plasma glucose levels drop in pregnancy between 6 and 12 WG, but not in women with a BMI >29.9 kg/m^2^ [19]. In our study too, BMI was a good predictor of GDM in women with FFPG ≥92 mg/dl. In a previous Italian multicenter study, we likewise found that prepregnancy BMI was an independent predictor of a complicated pregnancy in GDM women [20]. All these data confirm the importance of BMI in the development of gestational complications, and GDM in particular, underscoring the need to follow up overweight and obese women closely during pregnancy.

When maternal and fetal outcomes were considered in the present sample, we found no strong association between FFPG and adverse pregnancy outcomes. Riskin-Mashiah et al. reported that higher FFPG (even without reaching diabetic levels) increased the risk of LGA, macrosomia, and primary cesarean delivery [6]. Authors mentioned some limitations of their study, however, regarding differences in the glucose tests and screening tests used in the sample considered. This bias might explain the difference vis-à-vis our results in terms of pregnancy outcomes, as all the women in our study were tested using the same method and treated by a standardized method by a multidisciplinary team. More recently, also Sesmilo et al. demonstrated that FFPG is an independent predictor of GDM but also of LGA [21]. It should be noted that in this study authors used a different test to diagnose GDM (two step protocol with 100 g OGTT after an O'Sullivan test >140 mg/dl), and this can partly explain the different conclusions they reached. In our study we found that FFPG ≥92 mg/dl is a predictor of LGA only in NGT women and not in GDM women using the same test (OGTT 75 g) and the same criteria to diagnose GDM in all women. We can hypothesize that the early treatment in GDM women positively influenced fetal growth by reducing the frequency of LGA.

As regards the possible association between FFPG and the frequency of LGA, it is to underline that there are different growth curves to define LGA. In fact, in our study we used the standard Italian growth curves [12], Riskin et al. referred to growth curves of Israel [22], Sesmilo et al. haven't define the growth curves used for LGA definition. As for different tests used to diagnose GDM, the different growth curved used can partly explain the different results obtained in the studies.

In terms of pregnancy outcomes, we found that FFPG ≥92 mg/dl was associated with a different treatment for GDM. In fact, FFPG ≥92 mg/dl was a predictor of insulin need during pregnancy. This may mean that FFPG can predict the need for insulin therapy during pregnancy once GDM has been diagnosed. In previous studies, it was only the early diagnosis of GDM - not FFPG—that was found associated with a greater need for insulin treatment during pregnancy [23, 24]. So FFPG should be considered an early marker of insulin therapy once GDM has been diagnosed, underscoring the need to follow up women with an altered FFPG closely during pregnancy.

Our study has some strengths and some limitations. The main strengths are: the large sample studied, the standardized diagnosis and follow up of all GDM women. In particular, our study identifies the best cut-off level of FFPG tested in the first trimester for the prediction of GDM in a large population of pregnant women. The main limitation of the study is the retrospective nature of the study. In addition it is to underline that we studied a large Italian population and we used standard Italian growth curves to define LGA babies and these points prevent the generalization of the results to other populations.

In conclusion, we found that FFPG ≥92 mg/dl is related to GDM diagnosis and to the need of insulin therapy if GDM is diagnosed. An early diagnosis and a prompt start of insulin therapy are essential to prevent maternal and fetal complications. However, for standardizing the use of FFPG, prospective studies need to be conducted to define specific glucose cutoffs for each gestational week to improve its prediction of GDM development and of maternal and neonatal outcomes.

## Figures and Tables

**Table 1:** Clinical and metabolic parameters of the study sample, grouped by NGT versus GDM, and by FFPG (<92 mg/dl or ≥92 mg/dl).

	NGT <92 541	NGT ≥92 143	*P*1	GDM <92 470	GDM ≥92 283	*P*2	*P*3
Age (yrs)	33.3 ± 5.2	32.9 ± 5.6	0.541	34.6 ± 4.6	34.5 ± 4.9	0.802	0.004
BMI (kg/m^2^)	22.3 ± 4.0	24.3 ± 5.6	**<0.001**	23.7 ± 4.7	25.8 ± 5.4	**<0.001**	**<0.001**
Ethnicity (Italian) (%)	92.3	90.3	ns	89.7	87.5	ns	ns
Family history of diabetes (%)	10.2	9.6	ns	17.9	29.3	**<0.001**	**0.001**
Obesity (%)	3	4.1	ns	5	13.4	**<0.001**	**0.028**
Overweight (%)	6.1	7.1	ns	7.0	19.2	**<0.001**	0.044
Age >35 yrs (%)	26.2	14.4	**<0.001**	23.4	35	**<0.001**	0.022
Previous GDM (%)	0.8	1.2		3.1	9.1	**<0.001**	0.001
WG at FFPG	7.9 ± 1.4	7.9 ± 1.2	0.745	8.2 ± 2.4	7.7 ± 2.0	0.004	0.191
WG at diagnosis	na	na		25.2 ± 2.6	21.9 ± 6.0	**<0.001**	na
FFPG (mg/dl)	80.7 ± 6.0	95.3 ± 4.3	**<0.001**	82.4 ± 5.8	98.1 ± 6.5	**<0.001**	**<0.001**
Weight gain (kg)	11.7 ± 3.7	12.4 ± 3.3	0.521	9.2 ± 4.4	10.6 ± 6.1	0.468	0.380
Gestational hypertension (%)	1.7	2.2	ns	2.4	4.1	0.099	0.766
WG at delivery	39.7 ± 1.5	39.8 ± 1.6	0.537	38.9 ± 1.7	38.7 ± 1.6	0.402	**<0.001**
Birthweight (g)	3328 ± 500	3363 ± 537	ns	3273 ± 474	3249 ± 508	**0.521**	**0.031**
Macrosomia (%)	3.2	3.6	ns	2.2	2.6	ns	0.011
Neonatal hypoglycemia (%)	0.7	0.0	ns	1.3	3.8	ns	**<0.001**
Insulin therapy (%)	na	na		13.6	29.9	**<0.001**	na
LGA	2.5	4.1	ns	3.6	6.9	0.053	**0.013**
SGA	6.6	3.3	ns	4.7	7.2	ns	**0.013**

NGT, normal glucose tolerance; GDM, gestational diabetes mellitus; BMI, body mass index; WG, week of gestation; FFPG, first fasting plasma glucose test; ns = not significant; na = not applicable. Comparisons: *P*1, NGT <92 versus ≥92; *P*2, GDM <92 versus ≥92; *P*3 NGT ≥92 versus GDM ≥92.

**Table 2:** Multivariate analysis to identify predictors in early pregnancy of subsequent GDM based on first fasting plasma glucose levels <92 mg/dl or ≥92 mg/dl.

Predictors	Dependent variable: presence of GDM	
	Odds ratios (95%CI)	*pvalue*
First FPG (≥92)	1.924 (1.488–2.492)	**<0.001**
BMI	1.594 (1.231–2.067)	**<0.001**
Family history (yes)	2.480 (1.932–3.192)	**<0.001**
Age (≥35)	1.329 (1.059–1.670)	**0.014**
Previous GDM (yes)	4.100 (2.266–7.972)	**<0.001**

**Table 3:** Multivariate analysis to identify predictors of LGA in NGT (a) and GDM women (b).

Predictors	Odds ratios (95%CI)	*pvalue*
(a)		
*Dependent variable: LGA (NGT women)*		
First FPG (≥92)	2.340 (1.173–4.574)	**0.014**
BMI	2.634 (1.333–5.130)	**0.005**

(b)		
*Dependent variable: LGA (GDM women)*		
First FPG (≥92)	0.985 (0.562–1.707)	0.956
BMI	3.063 (1.768–5.338)	**<0.001**

## Data Availability

The data supporting the findings of this study are available within the article.
